# Comodulation of Dengue and Chikungunya Virus Infection During a Coinfection Scenario in Human Cell Lines

**DOI:** 10.3389/fcimb.2022.821061

**Published:** 2022-04-28

**Authors:** Debjani Taraphdar, Bharati Singh, Sabyasachi Pattanayak, Avula Kiran, Poornima Kokavalla, Mohd. Faraz Alam, Gulam Hussain Syed

**Affiliations:** ^1^Virus-Host Interactions Lab, Infectious Disease Biology, Institute of Life Sciences, Bhubaneswar, India; ^2^School of Biotechnology, Kalinga Institute of Industrial Technology, Bhubaneshwar, India; ^3^Regional Centre for Biotechnology, Faridabad, India

**Keywords:** antibody-dependent enhancement, coinfection, superinfection, dengue (DENV), chikungunya (CHIKV), liver

## Abstract

The Dengue virus (DENV) and Chikungunya virus (CHIKV) are the arboviruses that pose a threat to global public health. Coinfection and antibody-dependent enhancement are major areas of concern during DENV and CHIKV infections, which can alter the clinical severity. Acute hepatic illness is a common manifestation and major sign of disease severity upon infection with either dengue or chikungunya. Hence, in this study, we characterized the coexistence and interaction between both the viruses in human hepatic (Huh7) cells during the coinfection/superinfection scenario. We observed that prior presence of or subsequent superinfection with DENV enhanced CHIKV replication. However, prior CHIKV infection negatively affected DENV. In comparison to monoinfection, coinfection with both DENV and CHIKV resulted in lower infectivity as compared to monoinfections with modest suppression of CHIKV but dramatic suppression of DENV replication. Subsequent investigations revealed that subneutralizing levels of DENV or CHIKV anti-sera can respectively promote the ADE of CHIKV or DENV infection in FcγRII bearing human myelogenous leukemia cell line K562. Our observations suggest that CHIKV has a fitness advantage over DENV in hepatic cells and prior DENV infection may enhance CHIKV disease severity if the patient subsequently contracts CHIKV. This study highlights the natural possibility of dengue–chikungunya coinfection and their subsequent modulation in human hepatic cells. These observations have important implications in regions where both viruses are prevalent and calls for proper management of DENV-CHIKV coinfected patients.

## Introduction

Dengue and chikungunya infections are the most common mosquito-borne infections in India (Taraphdar et al., 2012). As per the National Vector Borne Disease Control Programme (NVBDCP), Government of India, more than 100,000 cases are reported annually due to dengue virus (DENV) and chikungunya virus (CHIKV) infections. An infection with either DENV or CHIKV can lead to self-limiting fever with a clinical presentation similar to febrile illness. However, in some dengue patients, severe dengue disease may manifest, leading to the lethal hemorrhagic fever or shock syndrome (Taraphdar et al., 2012; Beltrán-Silva et al., 2018). Debilitating polyarthralgia is a symptom unique to CHIKV infection, which may usually last for a few days but may be prolonged for weeks, months, or even years (Goupil and Mores, 2016). Infants, elderly, and people with comorbidities are at risk for more severe disease. In some patients, coinfection by these viruses has been reported. Both DENV and CHIKV viruses are transmitted by *Aedes* mosquitoes, and the regions of CHIKV prevalence often overlap with DENV-endemic areas (Myers and Carey, 1967; Taraphdar et al., 2012; Furuya-Kanamori et al., 2016; Kaur et al., 2018; Amraoui et al., 2019). Hence, there is a higher propensity of coinfection cases to occur in these regions.

The understanding of the clinical manifestations of dengue-chikungunya coinfection is very limited. The cocirculation and coinfection of DENV and CHIKV in patients is quite common and reported from several countries, including India (Myers and Carey, 1967; Taraphdar et al., 2012; Furuya-Kanamori et al., 2016; Kaur et al., 2018; Turuk et al., 2021). In Odisha, India, 30%–40% of dengue-chikungunya coinfection cases were reported in a hospital-based cross-sectional study in 2013 (Saswat et al., 2015). Some studies suggest that neither symptoms nor the clinical outcome was exacerbated by coinfection, whereas some report a high rate of severe symptoms and poor clinical outcome among coinfected patients (Taraphdar et al., 2012; Furuya-Kanamori et al., 2016). The varied observations among different studies highlight the need to understand the significance of dengue-chikungunya coinfection in other physiologically relevant human cells to determine the outcome of coinfection on disease pathogenesis. Both DENV and CHIKV affect the liver and the severity of acute liver dysfunction has been implicated as a marker for progression into severe dengue disease (Dalugama and Gawarammana, 2017; Oliveira et al., 2017). In addition to coinfection, the antibody-dependent enhancement (ADE) of infection may also lead to varied outcomes of disease manifestations. Dengue hemorrhagic fever and dengue shock syndrome are severe complications that have often been correlated with the ADE-mediated enhancement of secondary infection by the heterotypic dengue serotype (Guzman et al., 2013; Katzelnick et al., 2017). Interestingly, ADE is also observed in CHIKV infection (Lum et al., 2018) and in antigenically similar viruses like dengue and Zika (Wen and Shresta, 2019) resulting in the augmentation of virus infection by subneutralizing antibodies and the exacerbation of disease severity.

In this study, we focused on the modulation of DENV and CHIKV infection during a coinfection/superinfection scenario. We determined how a prior or current infection with either DENV/CHIKV impacts the outcome of subsequent infection with CHIKV/DENV in human hepatic Huh7 cell line and also investigated the possibility of the antibody (Ab)-mediated enhancement of DENV or CHIKV infections in Fc**γ**RII-bearing human myelogenous leukemia cells.

## Methods

### Cells, Reagents, and Antibodies

The African green monkey (*Cercopithecus aethiops*) kidney epithelial cell line Vero and human hepatoma cell line Huh7 were cultured in Dulbecco’s modified minimum essential medium (DMEM) supplemented with 10% (v/v) fetal bovine serum (FBS), 100 U/ml penicillin G, 100 μg/ml streptomycin, and non-essential amino acids (NEAAs) at 37°C with 5% CO_2_. Human FcγRII-expressing K-562 cells from a monocyte–granulocyte lineage with lymphoblast morphology (a kind gift from Dr. Soumen Chakraborty, ILS) were cultured in RPMI-1640 supplemented with 10% (v/v) FBS, 2 mM L-glutamine, 100 U/ml penicillin G, and 100 μg/ml streptomycin at 37°C with 5% (v/v) CO_2_. C6/36 mosquito cell lines were propagated in Leibovitz L-15 media at 28°C, supplemented with 10% heat-inactivated FBS, gentamycin, 100 U/ml penicillin G, 100 μg/ml streptomycin and 0.25 μg/ml amphotericin B, and 1× tryptose phosphate broth. All reagents were purchased from Gibco (United States). All cell lines were routinely checked for mycoplasma contamination and cultures free of contamination were used for experimentation.

### Viral Stocks

DENV serotype 2 isolate P23085 INDI-60 (GenBank Accession No. KJ918750.1) was a kind gift from Dr. Manjula Kalia, RCB, Faridabad, India. CHIKV S27 strain (GenBank Accession no AF369024.2) was obtained from the National Institute of Virology, Pune, India. The viruses were revived and propagated in the C6/36 mosquito cell line at a multiplicity of infection (MOI) of 0.1 till 72 h, and virus-containing supernatants were clarified by centrifugation at 2,000 rpm at 4°C before being stored at −80°C. Viral titers were determined by focus-forming unit assays in Vero cells.

### Virus Growth Kinetics and Quantitative Real-Time Polymerase Chain Reaction

Huh7 cells were infected with either CHIKV or DENV at MOI 1. Infected cells were incubated for 12, 24, 36, 48, 60, and 72 h postinfection and cells collected at respective time points. The cellular RNA was isolated using the Trizol reagent (Life Technologies, United States) as per the manufacturer’s protocol. Viral genome copies were measured by quantitative real-time polymerase chain reaction (qRT-PCR) using the Fast Virus RT-PCR Kit (Thermo Fisher) in Quant Studio 6 Real-Time PCR system, following the manufacturer’s protocol. CHIKV primers (2906F: 5’-CACGTCAACGTACTCCTAAC-3’ and 3038R: 5’-CCACCTCCCACTCCTTAATA-3’) and 2972 CHIKV probe (5’-Cy3)-TGGATAAAGACGCTGCAGAACCCA-(BHQ2-3’) and DENV primers (D2 FP 5’- CATAGGTATGGGCGTGACTTATC-3’ and D2 RP 5’- ATTCCTTGGAGGTCAGCTTT-3’) and D2 probe (5’-Fam- AGCAGCCTTCAAAGTCAGACCAACT-BHQ1-3’) were used. The relative quantification of viral copies was assessed using a standard curve. Standards were prepared by the quantitation of log-fold dilutions of a known quantity of the cloned plasmids harboring the genome segments targeted by the primer-probe sets. The plasmids were generated using the Infusion HD cloning kit (Takara, Kusatsu, Japan). **Table S1** shows the primers used for cloning.

### DENV-CHIKV Coinfection or Superinfection

Huh7 cell lines were infected as coinfection (mixed infection by both viruses), one infection followed by another after a specified time interval (superinfection) and single virus infection (monoinfection), as described previously (Zaidi et al., 2018). For superinfection experiments, the time gap between two infections was maintained as 12, 24, or 36 h, followed by superinfection for another 24 h. Cells were infected with DENV or/and CHIKV at MOI 1 for monoinfection, coinfection, and superinfection for 24 and 36 h. For experiments involving the pretreatment of naïve Huh7 cells with supernatants from DENV/CHIKV-infected cells, the collected supernatants were preclarified and subjected to Ultraviolet C irradiation (UVC) (254 nm) treatment for 5 min. The UV-treated or untreated supernatants obtained from DENV/CHIKV-infected cells were used for the pretreatment of the naïve Huh7 cells for 8 h, followed by infection with 1 MOI of virus (CHIKV or DENV), different than that in pretreatment culture supernatants. Approximately 24 h postinfection, the cells and cell-free supernatant were collected to determine the viral genome copies.

### Immunofluorescence for Cells With Dual Infection

Mock, CHIKV-infected, DENV-infected, and CHIKV-DENV coinfected or superinfected Huh7 cells, grown on glass coverslips, were washed with Phosphate Buffered Saline (PBS) and fixed with 4% paraformaldehyde at room temperature for 15 min. The monoinfected and coinfected cells were kept for 48 h, and for superinfection, a 24 h time interval was considered between the first and second infection. After washing thrice with PBS, the cells were blocked and permeabilized with 3% normal goat serum (Gibco Invitrogen) and 3% BSA (Sigma) in PBS with 0.1% Triton X-100 for 30 min. The cells were incubated with a mouse monoclonal anti-chikungunya virus (A54Q) Ab (Thermo Fisher Scientific, USA) and rabbit polyclonal dengue virus NS3 protein-specific Ab (GeneTex) diluted (1:1,500) in PBS with 3% BSA at 4°C overnight, followed by 3× wash with PBS containing 0.1% Tween 20. The cells were incubated at room temperature in the dark for 1 h with Alexa Fluor-conjugated donkey anti-mouse and anti-rabbit IgG antibodies (Life Technologies, Grand Island, NY, United States) diluted 1:500 in PBS, followed by 3× wash with PBS 0.1% Tween 20. The cover slips were mounted onto glass slides using the Prolong^®^ Gold antifade reagent (Invitrogen) and visualized under a Leica SP5 confocal microscope

### Focus-Forming Unit Assays

Vero cells were seeded at a density of ~10,000 cells in each well of a 96-well plate 24 h prior to infection. For titer assays, 10-fold serial dilutions of virus were prepared and used to infect the cell monolayers at 37°C. After 2 h, the inoculum was aspirated, cells washed with 1× PBS and incubated for further 48 (CHIKV) and 60 h (DENV). Foci were detected by incubating the 4% Paraformaldehyde (PFA)-fixed cells for 12 h at 4°C using a Chikungunya virus Ab (Thermo Fisher, United States) at 1:50 dilution or 1 μg/ml of mouse mAbs against DENV, followed by 3× wash with PBS and 1 h incubation at room temperature with 1:1,000 diluted Alexa Fluor-conjugated donkey anti-mouse IgG (H+L) (Thermo Fisher, USA). The foci were visualized using an Olympus DX58 fluorescent microscope.

### Antibody-Dependent Enhancement Assay

CHIKV IgM-positive patient sera S1 and S4 were obtained from RMRC, Bhubaneswar, India and DENV IgG-positive patient sera DenS were obtained from the S.C.B. Medical College, Cuttack, India. All the samples were monoinfected laboratory-confirmed cases and had no history of any another infection. The control serum (CS) was collected from healthy subjects without prior infection history. The sero-positive and sero-negative status of DENV and CHIKV patient sera was confirmed by DENV/CHIKV-specific ELISA and chikungunya-IgM and dengue-IgM and IgG rapid detection kits. Informed consent was taken in every case. Sera were heat-inactivated for 30 min at 56°C prior to use. FcγRII-expressing K562 cell lines were exposed to i) CHIKV at MOI 10 in the presence of either monoclonal antibodies (mAbs) to dengue virus 2 or dengue-patient sera and ii) DENV at MOI 10 in the presence of either of chikungunya-positive patient sera S1 and S4. In all cases, an equal volume of virus inoculum and Ab/serum dilutions were mixed and incubated at room temperature for 1.5 h for the formation of the virus–Ab complex. The dengue/chikungunya patient sera were diluted in DMEM at 1:50, 1:100, 1:200 and 1:500. The control sera were also diluted similarly. Preincubated virus and Ab mixtures were allowed to infect the cells for 2 h. After incubation, the cells were centrifuged at 2,000 rpm and the supernatant was discarded. The cells were washed with 1× PBS and incubated for 18 h with complete media. Viral genome copies were measured by qRT-PCR of total cellular RNA.

### Promoter Reporter Assay

Huh7 cells were seeded at 60%–80% confluency in a 6-well plate. The next day, the cells were cotransfected with a firefly luciferase based IFN-β promoter, IFN-γ promoter, and ISRE-luc reporter plasmids, respectively, along with a Renilla luciferase vector (used as transfection control to normalize transfection efficiency) for 16 h, followed by infection with 1 MOI of either CHIKV or DENV based on a monoinfection or superinfection scenario as described before. Approximately 36 h postinfection, the cell lysate were prepared in a 1× passive lysis buffer, and the Firefly and Renilla luciferase activities were determined using the dual-luciferase reporter system (Promega) according to the manufacturer’s instructions. The Firefly and Renilla luminescence was detected using the VICTOR Nivo multimode reader. The IFN-β promoter (Addgene No: 102597) and IFN-γ promoter (Addgene No: 17598) Firefly luciferase reporter plasmids were obtained from Addgene. The ISRE-promoter reporter plasmid was a kind gift from Dr. Aleem Siddiqui, University of California, San Diego (UCSD).

### Western Blotting

Cells were lysed with the addition of a radio-immunoprecipitation assay buffer in ice for 30 min. Cell lysates were centrifuged at 12,000 × g for 20 min at 4°C. The clarified supernatant protein concentration was determined using the Pierce BCA protein assay kit (Thermo Scientific, Waltham, MA, United States). The proteins were separated by electrophoresis and transferred to nitrocellulose membranes. Membranes were blocked for 1 h at room temperature using 5% bovine serum albumin in PBS-0.1%Tween 20, followed by overnight incubation at 4°C with the primary Ab against target proteins (anti-CD32; Santa Cruz Biotechnology, Dallas, TX, United States and in house antibodies against CHIKV E2). Blots were subsequently incubated with a 1:10,000 dilution of a horseradish peroxidase-conjugated secondary goat anti-rabbit IgG or goat anti-mouse IgG (Promega). The signals were detected using SuperSignal Chemiluminescent Substrate (Biorad, Herecules, CA, United States).

### Statistical Analysis

All the experiments were performed in two or three independent replicates, and the data shown are mean ± standard error of mean. Statistical analysis was performed using Student’s t-test or 2-way ANOVA as per the experimental requirement to determine the significance level (Graphpad Prism 5, United States). A p-value of ≤ 0.05 was considered significant.

### Ethical Statement

The protocol was approved by the Ethical Committee of Institute of Life Sciences, India (IEC/IRB-63/HEC/17).

## Results

### DENV Replication Is Suppressed in Presence of CHIKV; in Contrast, CHIKV Replication Is Enhanced in Presence of DENV

**Figure 1:**
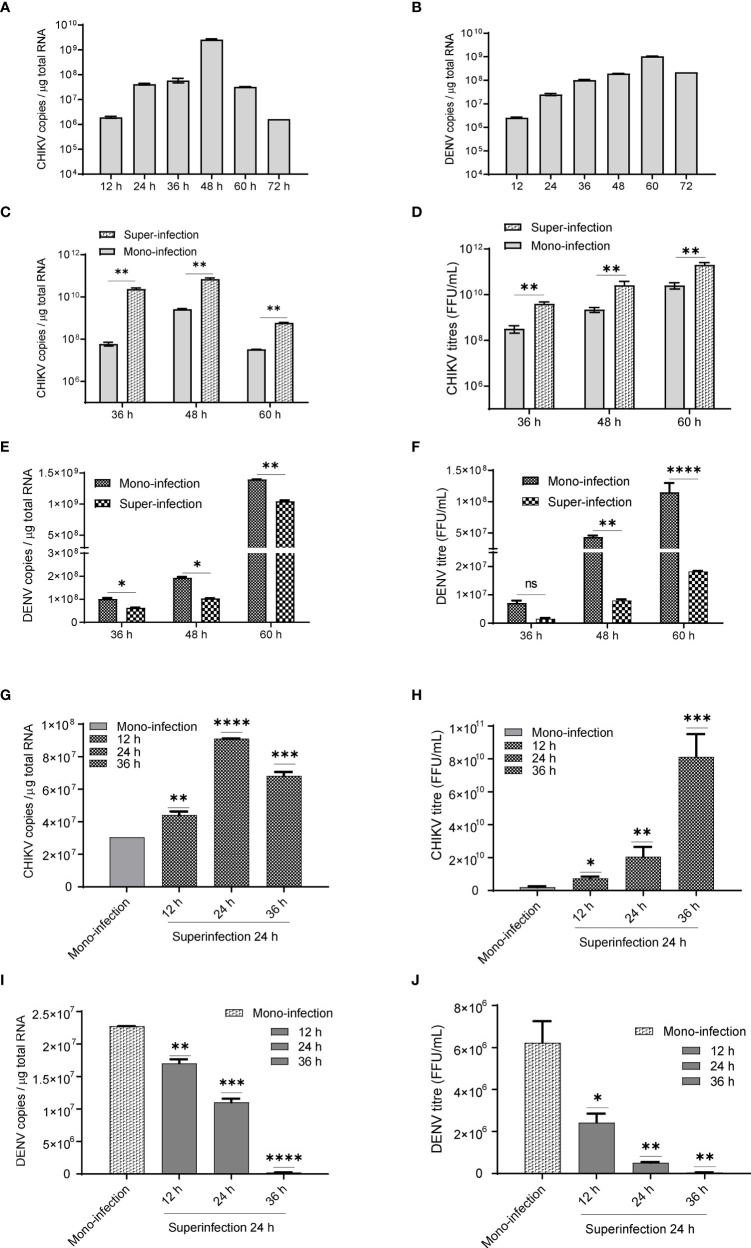
**(A, B)** Growth kinetics of CHIKV and DENV in Huh7 cell lines. Huh7 cells were infected with 1 MOI and viral genome copies estimated at 12, 24, 36, 48, 60, and 72 h postinfection. Bar graph depicting the CHIKV genome copies **(1A)** and DENV genome copies **(B)** per microgram of total cellular RNA. **(C, D)** CHIKV infection, followed by superinfection with DENV. CHIKV-infected Huh7 cells at 12, 24, and 36 h postinfection were subjected to superinfection with DENV for additional 24 h. The MOI of 1 was used for infection in all conditions. **(C)** The bar graph depicts the CHIKV genome copies in CHIKV-DENV-superinfected and CHIKV-monoinfected Huh7 cells at 36, 48, and 60 h postinfection. **(D)** Graphical representation of infectious CHIKV titers (FFU/ml) in the culture supernatant of CHIKV>DENV-superinfected and CHIKV-monoinfected Huh7 cells at 36, 48, and 60 h postinfection. **(E, F)** DENV infection followed by superinfection with CHIKV. DENV-infected Huh7 cells at 12, 24, and 36 h postinfection were subjected to superinfection with CHIKV for additional 24 h. An MOI of 1 was used for infection in all conditions **(E)**. The bar graph depicts the DENV genome copies in DENV-CHIKV-superinfected and DENV-monoinfected Huh7 cells for 36, 48, and 60 h postinfection. **(F)** Graphical representation of infectious DENV titers (FFU/ml) in the culture supernatant of DENV>CHIKV-superinfected and DENV-monoinfected Huh7 cells for 36, 48, and 60 h postinfection. **(G, H)** The bar graph depicts CHIKV genome copies **(G)** and CHIKV titers (FFU/ml) **(H)** after 24 h post CHIKV-infection in naïve Huh7 cells or cells already harboring DENV for 12, 24, and 36 h. **(I, J)** The bar graph depicts DENV genome copies **(I)** and DENV titers (FFU/ml) **(J)** after 24 h post-DENV infection in naïve Huh7 cells or cells already harboring CHIKV for 12, 24, and 36 h. All the experiments were performed as three independent replicates. The data represented are mean of the replicates ± SEM (*P < 0.05, **P < 0.01, ***P < 0.001, ****P < 0.0001, ns, non-significant).

**Figure 2:**
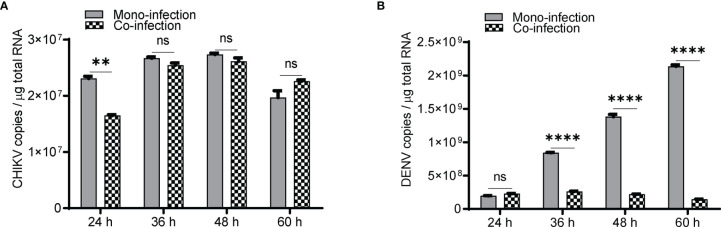
Coinfection (simultaneous) of Huh7 cells with CHIKV and DENV. Naïve Huh7 cells were simultaneously (coinfected) infected with 1 MOI of both CHIKV and DENV or monoinfected with CHIKV and DENV, respectively. Bar graph depicting the CHIKV genome **(A)** and DENV genome **(B)** copies in coinfection vs. monoinfection at respective time points postinfection. All the experiments were performed as three independent replicates. The data represented are mean of the replicates ± SEM. (**P < 0.01, ****P < 0.0001, ns, non-significant).

In Huh7 cells, CHIKV replication increases till 48 h (**Figure 1A**), whereas DENV replication increases up to 60 h postinfection (**Figure 1B**) in agreement with the growth kinetics reported by other groups (Ruiz Silva et al., 2017) suggesting that the CHIKV growth kinetics is more rapid and shorter than DENV. In CHIKV-infected cells, followed by DENV superinfection at 12, 24, and 36 h post infection, the introduction of DENV-enhanced CHIKV replication in comparison to CHIKV monoinfection for the same time (**Figure 1C**). An estimation of CHIKV infectious titers by the foci-forming unit assay also confirmed the release of more virus particles into the culture supernatants upon dengue superinfection in comparison to monoinfection (**Figure 1D**). In contrast, DENV replication was inhibited upon superinfection with CHIKV at 12, 24, and 36 h intervals in comparison to DENV monoinfection (**Figure 1E**), and the estimation of DENV infectious titers also validated the same (**Figure 1F**). In this experiment, the estimation of viral genome copies and infectious titers of the viruses used for superinfection suggested that the prior presence of DENV enhanced CHIKV replication in comparison to CHIKV monoinfection (**Figures 1G**, **H**). In sharp contrast, the prior presence of CHIKV infection led to inhibition in DENV replication upon superinfection with DENV in comparison to DENV monoinfection and the inhibition was higher with an increase in time interval (12, 24, and 36 h) between CHIKV infection and DENV superinfection (**Figures 1I, J**). This suggests that the presence of DENV promoted CHIKV replication, whereas the presence of CHIKV is inhibitory to DENV replication (**Figures 1G–J**). When the infection with both viruses was done simultaneously, as a coinfection scenario, we do observe a slight but non-significant suppression in CHIKV replication (**Figure 2A**) but dramatic suppression of DENV replication (**Figure 2B**) compared to the monoinfection at respective time points postinfection. Overall, these observations suggest that in liver epithelial cells, i) CHIKV has a fitness advantage compared to DENV, ii) prior DENV infection enhances CHIKV replication, and in contrast, prior CHIKV infection inhibits DENV replication in a superinfection scenario, and iii) simultaneous infection (coinfection) with both CHIKV and DENV suggests that CHIKV overpowers DENV.

### Coexistence of CHIKV and DENV in Hepatic Cells After Coinfection (Simultaneous) and/or Superinfection

**Figure 3:**
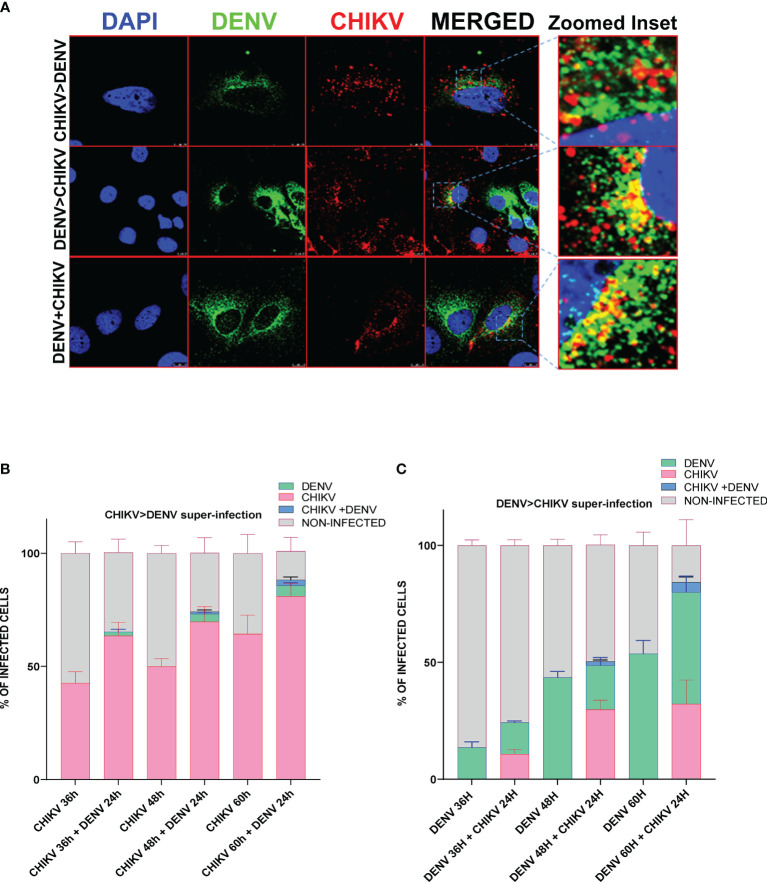
Immunofluorescence imaging of Huh7 cells monoinfected or superinfected with CHIKV and DENV. Huh7 cells were subjected to CHIKV and DENV coinfection for 48h, CHIKV or DENV monoinfection for 36, 48, and 60 h or superinfected with CHIKV/DENV for 24 h in cells already infected with DENV/CHIKV for 12, 24, and 36 h. An MOI of 0.5 was used throughout the experiment. The cells were fixed in 4% paraformaldehyde at respective time points postinfection and immunostained for DENV (green) and CHIKV (red). Images were taken in Leica SP5 confocal microscope. The nuclei were counterstained with DAPI. **(A)** Confocal images of Huh7 cells harboring both DENV (green channel) and CHIKV (red channel) in the scenario of coinfection or superinfection. The zoomed inset shows the cells harboring both the viruses. **(B, C)** Bar graph depicting the percentage of Huh7 cells infected with only CHIKV or DENV, or both CHIKV and DENV or uninfected cells in CHIKV monoinfection vs. CHIKV, followed by DENV superinfection scenario **(B)** or DENV monoinfection vs. DENV, followed by CHIKV superinfection scenario **(C)**. The experiments were done in three independent replicates, and the data shown are mean ± SEM.

To determine if both viruses can infect the same cells and to estimate the percentage of cells harboring both viruses, we performed immunofluorescence microscopy in CHIKV and DENV-coinfected and -superinfected Huh7 cells. In monoinfection and coinfection (simultaneous infection with both viruses), cells were infected with 0.5 MOI for 48 h and fixed with 4% PFA, whereas in the case of superinfection scenario, the cells were infected with 0.5 MOI for 12, 24, and 36 h and subsequently superinfected with 0.5 MOI of the other virus for additional 24 h. The confocal imaging showed the presence of both DENV and CHIKV proteins in the same cell in a very small percentage of cells infected by the superinfection and/or coinfection approach, suggesting that both the viruses can replicate in a single cell (**Figure 3A**), however, at a very low frequency as only a minor fraction of cells were observed to harbor both the viruses (**Figures 3B, C**). We then analyzed in respective monoinfection and CHIKV >DENV or DENV >CHIKV superinfection scenario the percentage of cells infected with only CHIKV or DENV, or both CHIKV and DENV or uninfected (**Figures 3B, C**). Approximately 7 image panels with 60–80 cells per image were analyzed for each condition. The CHIKV-infected cells at 12, 24, and 36 h postinfection, followed by 24 h DENV superinfection, displayed a higher number of CHIKV-infected cells in comparison to CHIKV-monoinfected cells at 36, 48, and 60 h postinfection, respectively (**Figure 3B**). Overall, these observations are strongly correlated with the viral replication data, suggesting that CHIKV replication is enhanced upon a dengue superinfection (**Figures 1C, D**). In contrast, DENV-infected cells at 12, 24, and 36 h postinfection, followed by 24 h CHIKV superinfection, displayed a lower number of dengue-positive cells compared to DENV monoinfection at 36, 48, and 60 h postinfection (**Figure 3C**), which is strongly correlated with a reduction in DENV replication levels upon superinfection with CHIKV (**Figures 1E–F**). Prior infection of DENV also promoted CHIKV infection evident by a higher percentage of CHIKV-infected cells in cells already harboring DENV infection for 24 and 36 h compared to those harboring DENV for 12 h (**Figure 3C**). In contrast, we observed a very minimal number of cells infected with DENV in a CHIKV-DENV superinfection scenario in agreement with a previous observation that the prior presence of CHIKV is inhibitory to subsequent DENV infection (**Figure 3B**).

### Treatment of Naïve Huh7 Cells With Supernatants From DENV/CHIKV Infected Cells Affected CHIKV/DENV Replication

**Figure 4:**
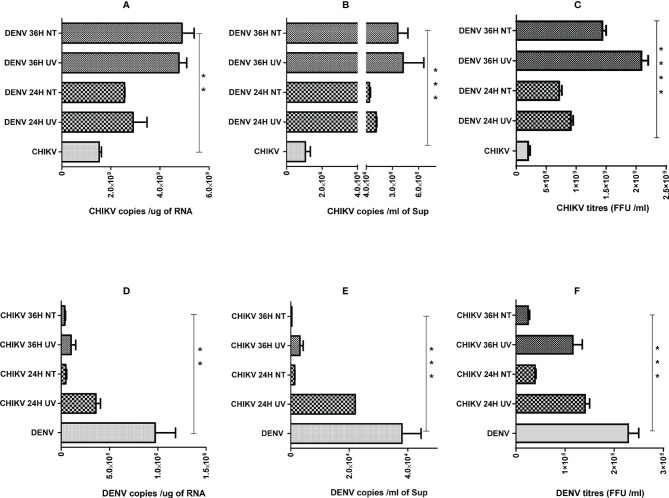
Effect of pretreatment with culture supernatants (sups) from DENV- or CHIKV-infected cells on CHIKV or DENV infection. Naïve Huh7 cells were pretreated for 8 h with clarified UV-treated or untreated sups obtained from DENV- or CHIKV-infected cells at 24 or 36 h post-infection. After respective pretreatment, the cells are infected with 1 MOI of CHIKV or DENV for 24 h. **(A–C)** Bar graph depicting the CHIKV genome copies in total cellular RNA **(A)**, CHIKV genome copies per milliliter of culture supernatant **(B)** and infectious CHIKV titers per milliliter of culture supernatant. **(D–F)** Bar graph depicting the DENV genome copies in total cellular RNA **(D)**, DENV genome copies per milliliter of culture supernatant **(E)**, and infectious DENV titers per milliliter of culture supernatant **(F)**, and infectious DENV titers per milliliter of culture supernatant. All the experiments were in done in three independent replicates, and the data shown are mean ± SEM. (****P < 0.0001, ***P < 0.001, **P < 0.01).

Since we observed that the percentage of cells harboring both the viruses was very low (**Figure 3**), we wanted to address if the effect of coinfection/superinfection on the modulation of either CHIKV or DENV replication is due to the extracellular signaling effect from the infected cells on the bystander naïve cells. For this, we treated naïve Huh7 cells for 8 h with UV-treated or untreated culture supernatants (sups) obtained from DENV- or CHIKV-infected cells prior to infection with 1 MOI of CHIKV or DENV, respectively. The replication status of DENV or CHIKV in Huh7 cells whose culture sups were used for pretreatment is shown in **Figures S1A, B**. UV treatment led to a substantial decline in the infectious titers of CHIKV or DENV in the culture sups as evident by the reduced level of infectious virus titers estimated by the FFU assay (**Figure S2A**). The infection of the naive cells with the untreated and UV-treated culture supernatants also yielded a low intracellular viral genome in cells infected with UV-treated culture supernatants, respectively (**Figure S2B**), further validating that UV treatment led to a reduction in infectious virus titers. In correlation to the results observed in a superinfection scenario, we observed that the pretreatment of naïve cells with both UV-treated and untreated culture sups obtained from DENV-infected Huh7 cells promoted CHIKV replication, leading to higher levels of intracellular (**Figure 4A**) and extracellular (**Figure 4B**) viral genome copies and infectious virus titers (**Figure 4C**). Surprisingly, both UV-treated and untreated culture supernatants had a similar effect, suggesting that the factors released into the culture supernatant from DENV-infected cells is sufficient to promote CHIKV infection and does not require the presence of the infectious dengue virus. The pretreatment of Huh7 cells with UV-treated or untreated culture supernatants obtained from CHIKV-infected Huh7 cells prior to DENV infection resulted in a dramatic inhibition of DENV replication evident by the low levels of intracellular (**Figure 4D**) and extracellular (**Figure 4E**) viral genome copies and infectious virus titers (**Figure 4F**). Interestingly, the inhibition was more profound in the cells pretreated with untreated compared to UV-treated culture sups, suggesting that the presence of the infectious CHIKV virus contributes to a greater inhibition of DENV replication.

### Antibody-Mediated Enhancement of CHIKV/DENV Infection With Subneutralizing Concentration of DENV/CHIKV Antisera or Antibodies

**Figure 5:**
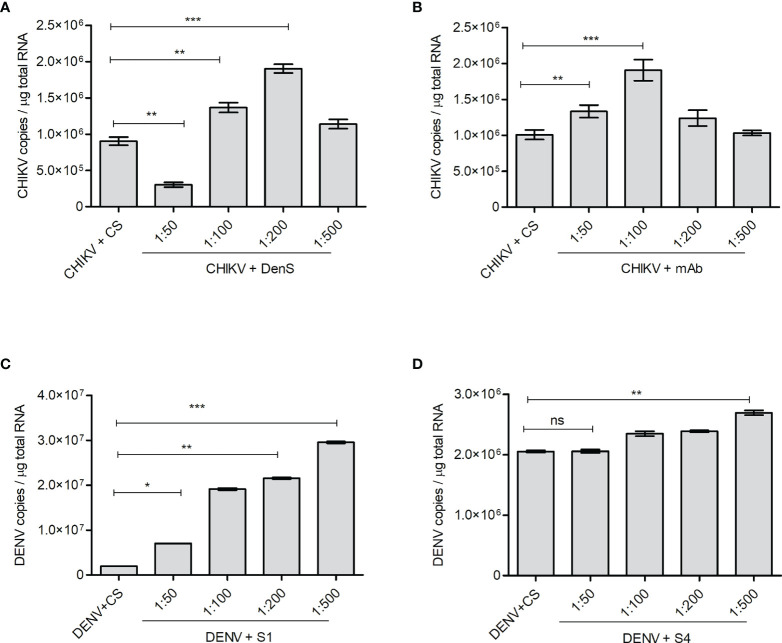
ADE of CHIKV or DENV infection. The K562 cells were infected with preincubated mixtures of CHIKV and DENV-specific mAb or dengue-patient serum DenS or preincubated mixtures of DENV and CHIKV-patient sera S1 and S4 at various dilutions as indicated. Serum of healthy volunteers (CS) at 1:200 dilution was used as control. Approximately 18 h postinfection, the level of CHKV/DENV viral RNA inside the K562 cells was estimated by qRT-PCR of total cellular RNA. **(A, B**) Bar graph depicting the CHIKV genome copies in K562 cells infected with preincubated mixtures of CHIKV and DENV patient serum DenS or DENV-specific mAb at indicated dilutions. **(C, D)** Bar graph depicting DENV genome copies in K562 cells infected with preincubated mixtures of DENV and CHIKV patient sera S1 or S4 at indicated dilutions. All the experiments were done in three independent replicates and the data shown is mean ± SEM (ns, non significant, *P < 0.05, **P < 0.01, ***P < 0.001).

The K562 cells bearing FcγRII are not highly supportive of CHIKV and DENV replication, although the cells get infected by both viruses. However, both DENV and CHIKV commonly showed FcγRII-mediated Ab-dependent enhancement; hence, K562 cells were used for this study (Chawla et al., 2013; Lum et al., 2018). As the K562 cells do not support active viral replication, the intracellular viral load in K562 cells starts to decline 24 h postinfection with either CHIKV or DENV. Hence, to investigate if the subneutralizing concentrations of DENV-specific or CHIKV-specific sera/antibodies promote the infection of K562 cells with CHIKV or DENV, respectively, the virus–Ab/anti-sera complex was allowed to infect for 2 h, followed by incubation for further 18 h, subsequently followed by the quantification of viral RNA by the qRT-PCR of total cellular RNA. The DenS serum dilution of 1:100 and 1:200 significantly promoted CHIKV uptake within the K562 cells (**Figure 5A**). Similarly, CHIKV infection was also enhanced with preincubation with DENV serotype 2-specific mAb HB46 (**Figure 5B**). DENV infection was also significantly enhanced in the K562 cells when preincubated with various dilutions of CHIKV patient sera S1 (**Figure 5C**); on the other hand, no enhancement was observed with patient sera S4 at lower dilution and only a slight increase was observed at 1:500 dilution. (**Figure 5D**). The Fc gamma receptor (FcγR)-mediated pathway is the most common mechanism in the ADE of infections (Lum et al., 2018). The DENV ADE is correlated with increased viremia and severe disease outcomes in FcγR-bearing cells including macrophages, monocytes, and dendritic cells. We wanted to correlate the enhanced virus entry due to ADE with the expression status of FcγRII; hence, we performed a Western blot analysis of cells subjected to infection with the virus (CHIKV/DENV) preincubated with various dilutions of Ab/antisera (**Figure S3**). We did not notice any significant change in the expression of FcγRII upon the incubation of K562 cells with the complexes of CHIKV with various dilutions of DenS/DENV-specific mAb used for the ADE experiment (**Figure S3**). Similarly, the treatment of K562 cells with complexes of DENV with various dilutions of the CHIKV patient sera did not result in a significant change in the expression of FcγRII (**Figure S3**). Overall, these observations suggest that the subneutralizing concentration of DENV or CHIKV patient sera or Abs can promote the ADE of a CHIKV or DENV infection, respectively.

### Analyses of Immune Response in the DENV/CHIKV Mono- and Superinfections

**Figure 6:**
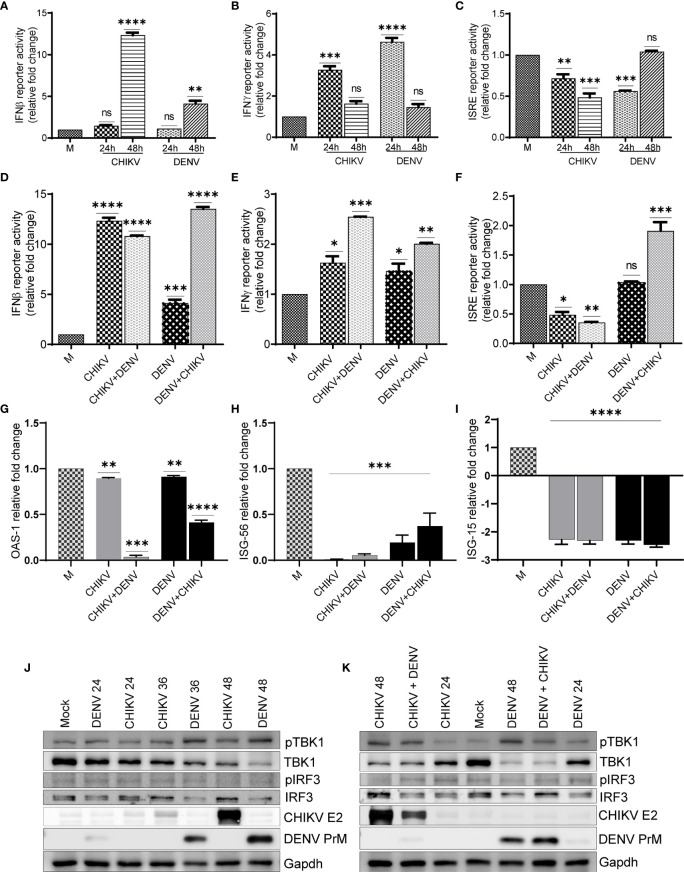
Activation status of antiviral signaling in monoinfection and superinfection scenario. Huh7 cells were transfected with IFNβ-luc, IFNγ-luc, and ISRE-luc reporter plasmids, respectively, for 16 h, followed by infection with 1 MOI of either CHIKV or DENV for 24 and 48 h. **(A–C)** Graph depicting IFNβ-luc activity **(A),** IFNγ-luc activity **(B),** and ISRE-luc activity **(C)** in either CHIKV- or DENV-infected Huh7 cells at respective time-point postinfection. **(D–F)** Huh7 cells transfected with IFNβ-luc, IFNγ-luc, and ISRE-luc reporter plasmid for 16 h, followed by infection with 1 MOI of CHIKV or DENV for 24 h and subsequently superinfected with or CHIKV for additional 24 h. Graph depicting IFNβ-luc activity **(D)**, IFNγ-luc activity, **(E)** and ISRE-luc activity **(F)** in either CHIKV/DENV monoinfection or superinfection. **(G–I)** Bar graph depicting fold changes in the transcript levels of OAS-1 **(G)**, ISG-56, **(H)** and ISG-15 **(I)** in the above cells. **(J, K)** Huh7 cells were infected with 1 MOI of either CHIKV or DENV for 24, 36, and 48 h, respectively **(J)**, or infected with 1 MOI of CHIKV or DENV for 24 h and subsequently superinfected with 1 MOI of DENV or CHIKV for additional 24 h **(K)**. Cell lysates obtained from these cells were subjected to Western blot analysis with indicated Abs. Abs targeting CHIKV envelope 2 and DENV PrM were used as infection markers, and Glyceraldehyde-3-phosphate Dehydrogenase (GAPDH) was used as loading control. All the experiments were done in three independent replicates, and the data shown are mean ± SEM (**** P < 0.0001, ***P < 0.001, **P < 0.01, *P < 0.05, ns, non-significant).

In order to depict whether the antiviral immune responses are specifically elicited/modulated by CHIKV or DENV monoinfection or during the condition of subsequent superinfection, we analyzed the promoter activity of interferons β and γ and interferon-stimulated response element (ISRE) present in the interferon-stimulated genes (ISGs). We also performed the activation/expression analysis of interferon regulatory factors (IRFs), their upstream activator Tank binding kinase 1 (TBK1), and ISGs. Our observation suggests that in comparison to mock-infected Huh7 cells, there was a significant increase in the IFNβ promoter activity in both DENV- and CHIKV-monoinfected cells at 48 h postinfection. Interestingly, the upregulation was several folds higher in CHIKV-infected cells compared to DENV-infected cells (**Figure 6A**). In the case of the superinfection scenario, the superinfection of CHIKV-infected cells with DENV resulted in a modest decrease in IFNβ promoter activity, whereas the superinfection of DENV-infected cells with CHIKV resulted in a substantial increase in IFNβ promoter activity (**Figure 6D**). These observations suggest that CHIKV infection, either as monoinfection or superinfection, sharply triggers IFNβ promoter activity in contrast to DENV infection (**Figures 6A, D**). In addition, we observed that both CHIKV and DENV induced IFNγ promoter activity at 24 h postinfection, although there was a sharp decline in the IFNγ promoter activity at a later time postinfection in both DENV- and CHIKV-infected cells (**Figure 6B**). Surprisingly, the case of superinfection with either DENV or CHIKV significantly triggered IFNγ promoter activity in comparison to the respective monoinfections (**Figure 6E**).

A monoinfection with either CHIKV or DENV did not trigger ISRE activity; however, we observed a gradual decline with time in CHIKV-infected cells compared to a mock infection (**Figure 6C**). In contrast, we observed a non-significant but slight increase in ISRE activity in DENV-infected cells at 48 h postinfection in agreement with an increase in IFNβ promoter activity observed at a later time postinfection (**Figure 6C**). The superinfection of CHIKV-infected cells with DENV did not lead to any increase in the ISRE activity; however, the superinfection of DENV-infected cells with CHIKV led to a modest increase in ISRE activity (**Figure 6F**). In agreement with ISRE activity, we did not find any induction in the expression of a few of the ISGs tested, OAS1 (2’-5’oligoadenylate synthetase1), ISG56, and ISG15 (interferon-stimulated genes 56 and 15) (**Figures 6G–I**). ISG-15 has a potential antiviral activity; we observed several fold decreases in the transcript level of ISG-15 in both monoinfection and superinfection cases (**Figure 6I**). It has been previously reported that both DENV and CHIKV inhibit interferon signaling through distinct mechanisms (Fros et al, 2010; Rodriguez-Madoz et al, 2010; Göertz et al., 2018; Kao et al., 2018; Uno and Ross, 2018), which is in agreement with our observations, suggesting a decline in ISRE activity and reduced expression of the respective ISGs analyzed.

Western blot analysis suggests a gradual increase of TBK1 kinase activity in both CHIKV and DENV-infected cells; however, the DENV-infected cells displayed higher TBK1 phosphorylation compared to CHIKV-infected cells (**Figure 6J**, and **Figure S4**). Despite the increase in TBK1 phosphorylation, both CHIKV and DENV infections led to a gradual decline in the TBK1 protein level during the course of infection, with the decline being higher in DENV-infected cells compared to CHIKV-infected cells (**Figure 6J** and **Figure S4**). The phosphorylation status of IRF3, the downstream target of TBK1, showed a slight increase in DENV-infected cells but no change in CHIKV-infected cells (**Figure 6J** and **Figure S4**). However, the DENV infection resulted in a gradual decline in the IRF3 protein levels with the time postinfection (**Figure 6J** and **Figure S4**). Superinfection with either DENV or CHIKV both led to a slight decrease in TBK1 phosphorylation and TBK1 protein levels in comparison to their respective monoinfections (**Figure 6K**). Similar to a monoinfection, we did not observe any significant increase in IRF3 phosphorylation in superinfected cells and the total IRF3 protein levels were also reduced upon superinfection with DENV (**Figures 6J, K**).

## Discussion

Viral interference is a common phenomenon where one virus competitively modulates the replication of other coinfecting viruses and is the most common outcome of coinfections. This can result in varied virulence and virus-mediated cytopathy, thereby affecting the disease severity. CHIKV, DENV, and ZIKV are all transmitted by *Aedes* mosquitos and also have a lot of overlap in the seasonal and geographical prevalence. Hence, there is a very high propensity of coinfection cases (Taraphdar et al., 2012; Göertz et al., 2017; Carrillo-Hernández et al., 2018). Both CHIKV and DENV affect many states in India every year and have always been a health burden with high morbidity and mortality (Source: NVBDCP). Although CHIKV-DENV superinfection cases are increasing at alarming rate in India (8.7%–15.05% cases) (Taraphdar et al., 2012; Saswat et al., 2015; Kaur et al., 2018), our understanding of the DENV-CHIKV interaction and the resultant outcome of this interaction is very limited. In virology, coinfection is used to describe the simultaneous infection of a cell or organism by different viruses, whereas superinfection is used if one virus infects the host sometime after infection by the first virus (Salas-Benito and De Nova-Ocampo, 2015). In this study, we attempted to decipher how the two highly prevalent arboviruses, DENV and CHIKV, modulate each other at the cellular level. The manifestation of acute hepatic illness is of common occurrence during the infection with CHIKV or DENV, and acute liver dysfunction is among the most common warning signs for the onset of severe dengue disease (Hadinegoro, 2012; Khandia et al., 2018; Manish et al., 2020). Hence, we conducted this study in the human hepatoma cells to be able to extrapolate these observations to the clinical context of coinfections.

An earlier study suggests that CHIKV proteins appear faster than DENV proteins postexposure in both midgut and salivary glands of the *Aedes* mosquito (Le Coupanec et al., 2017). Similarly, the studies conducted by Zaidi et al. (2018) suggest high CHIKV titers and low DENV titers in the coinfected Vero cells and in the coinfected Mexican infants (Zaidi et al., 2018). Another study done in the PBMCs reported that CHIKV exhibits faster growth kinetics than DENV, but in a coinfection scenario, the DENV replication was enhanced (Ruiz Silva al., 2017). Similarly, in coinfected mosquitos, DENV had a higher replication rate than CHIKV in salivary glands (Le Coupanec et al., 2017). A competitive suppression of CHIKV by higher titer of DENV in C6/36 cell line was reported by Potiwat et al. (2011). All these observations suggest that the modulation of coinfections may be specific to the host cell types involved. In this study using hepatic cell lines, we do observe that CHIKV exhibits faster growth kinetics than DENV and coinfection results in higher CHIKV titers than DENV, similar to that observed in Vero cells (Zaidi et al., 2018). However, the outcome of coinfection or superinfection was different from that observed in human PBMCs (Ruiz Silva et al., 2017) further confirming that the outcome of comodulation by either virus during coinfection or superinfection scenario may be cell type specific.

Superinfection exclusion is a phenomenon where a prior viral infection prevents a secondary infection (Folimonova, 2012). This may likely be due to the heightened antiviral state of the infected cells or viral gene expression (non-interferon mediated), resulting in a virus-induced cellular state of resistance to a subsequent viral infection. Competition may exist for the metabolites and cellular membrane/replication sites and can interfere at various stages of the viral life cycle, such as entry, genome replication, and viral particle egress (Kumar et al., 2018). Interestingly, both DENV and CHIKV subvert the host cell innate immune response through targeting multiple aspects of innate immune signaling (Göertz et al., 2018; Kao et al., 2018; Uno and Ross, 2018). Can the modulation of host immune response by the already resident viruses make the cells susceptible/non-susceptible to superinfection with a subsequent pathogenic virus? The ISRE activity and ISG expression status and downregulation of TBK1 and IRF3 in DENV and CHIKV infected cells confirm that both CHIKV and DENV modulate the host interferon signaling (**Figure 6**). However, our observations also suggest that the frequency of dual-infected cells is very low (**Figure 2**), which may be due to higher levels of infection-associated cytopathy or non-conducive intracellular environment in dual-infected cells. This indicates that the effect of DENV/CHIKV on subsequent superinfection with either CHIKV or DENV (**Figure 1**) is majorly mediated by factors emanating from the infected cells that affect the bystander naïve cells (**Figure 3**). However, more thorough investigations are required to identify these extracellular mediators. We are in the pursuit of performing global proteomic, transcriptomic, and metabolomic analyses in the DENV/CHIKV monoinfection and superinfection conditions to characterize the gene networks exclusively modulated during either scenarios.

The antibody-mediated enhancement (ADE) of DENV infection is shown to exacerbate disease severity (Chawla et al., 2013; Guzman et al., 2013). Pre-existing antibodies (Abs) present in the body from a primary DENV infection can bind to an infecting DENV particle during a subsequent infection with a different serotype and facilitate uptake in Fc gamma receptor (FcγR)-bearing immune cells (Chan et al., 2011; Gana et al., 2017). Zika virus (ZV) has emerged in dengue-endemic areas, and the Abs against DENV were found to enhance ZV infection (Dejnirattisai et al., 2016; Castanha et al., 2017; Langerak et al., 2019). Similarly, the ADE of CHIKV infection is also reported in *in vitro* and *in vivo* mouse models (Lum et al., 2018). The ADE by antibodies targeting different yet closely related viruses raises concerns over vaccination strategies and ADE-associated enhanced disease severity across viruses sharing antigenic resemblance. For instance, several reports suggest a substantial cross-reaction of DENV mABs or immune sera toward ZIKV (Spaeth et al., 2016; Langerak et al., 2019). Due to the increasing prevalence of CHIKV in DENV endemic areas and increasing rates of coinfection, we investigated if CHIKV/DENV anti-sera/antibodies promote the ADE of DENV/CHIKV infection at subneutralizing concentrations. Surprisingly, we observe that the subneutralizing concentration of the immune sera showed a significant enhancement of infection of CHIKV/DENV viruses in K562 cells (**Figure 5**). ADE-mediated infection involves the entry of virus–Ab complexes into the immune cells *via* Fcγ receptors (Rodrigo et al., 2006; Chan et al., 2011; Boonnak et al., 2013). We used K562 cells that express FcγRII (Chiofalo et al., 1988; Rodrigo et al., 2006; Rosales, 2017) to evaluate the involvement of ADE in DENV and CHIKV infection as FcγRII is reported to be more effective than FcγRI in mediating ADE by the immune complexes of either DENV or CHIKV (Le Coupanec et al., 2017; Lum et al., 2018). Although CHIKV and DENV belong to a different family of RNA viruses, the ADE exhibited by DENV/CHIKV anti-sera and antibodies may be due to the structural mimicry between the antigenic epitopes of DENV and CHIKV envelope proteins. In agreement, Kam et al. (2015) observed that serum samples from flavivirus-infected patients showed a low level of cross-reactivity against E2EP3, part of a CHIKV protein (Kam et al., 2015) In addition, the profound levels of glycosylation usually noticed on viral envelope glycoproteins can also lead to cross-neutralization and cross-reactivity due to the structural similarity of the glycans present on viral glycoproteins.

It is typically considered that viral infections lead to type I and type III interferon-mediated antiviral signaling and subsequent upregulation of the putative ISGs (Ivashkiv and Donlin, 2014, Megan et al., 2020). Although we observe the upregulation of IFNβ promoter activity at later stages of DENV or CHIKV monoinfection, we do not observe a concomitant upregulation of ISRE activity (**Figure 6**). Superinfection were also associated with similar alteration in promoter activity as observed in respective monoinfection. We observed the downregulation of IFNγ, ISG15, and OAS1 in both the mono- and superinfection scenario (**Figure 6**). ISG15 is a potent antiviral and has been shown to restrict many viruses at various stages of viral life cycle including dengue; therefore, the downregulation of ISG15 may be a viral strategy to evade host immune defense (Hishiki T. et al., 2014; Perng and Lenschow, 2018). Our analysis did not reveal any major clues as to how these viruses may modulate the infection of each other in a superinfection scenario in the context of antiviral signaling. This may be due to the cell type, Huh7, used in this study, which does not stage a strong immune response upon the challenge with viruses unlike the cells of myeloid lineage. A recent study on cell type-specific interferon response suggests that Huh7 cells do not show any differential expression in interferon signaling upon challenge with SARS-CoV-2 (Saccon et al., 2021). Interestingly, we do observe that DENV downregulates the expression of TBK1 and IRF3, the two major players in the Retinoic acid-inducible gene I (RIG-I)-Mitochondrial antiviral-signaling (MAVS) axis of antiviral signaling. This may be one of the many reasons why superinfection or prior infection of DENV promotes CHIKV growth (**Figure 1**). However, this may be only feasible in cells harboring both the viruses, which are quite few in number and may not significantly contribute to the overall observations made. A holistic multiomic analysis is required to characterize and identify the distinct molecular mechanisms that differentially affect the outcome of monoinfection and superinfection.

Overall, our study demonstrates that DENV and CHIKV modulate the replication of each other and CHIKV has a fitness advantage over DENV in hepatic cells. These observations highlight that prior DENV infection and subsequent CHIKV infection may have detrimental consequence on liver health and might have major implications in driving severe dengue or CHIKV pathogenesis. This study implicates the dangers associated during a coinfection scenario and suggests proper management of DENV-CHIKV-coinfected patients as the presence of either virus can affect the disease severity by the modulation of viral propagation.

## Data Availability Statement

The original contributions presented in the study are included in the article/**Supplementary Material**. Further inquiries can be directed to the corresponding author.

## Ethics Statement

The studies involving human participants were reviewed and approved by Ethical Committee of Institute of Life Sciences, India. The patients/participants provided their written informed consent to participate in this study.

## Author Contributions

GS revised and edited the manuscript. DT and GS conceived the idea, analyzed the data, and prepared the manuscript. DT and BS performed data curation. DT, BS, and GS performed the formal analysis, visualization, and validation of the data. DT, BS, SP, AK, MFA, and PK performed investigation. All authors read and made the final approval of the manuscript.

## Funding

Intermediate fellowship (IA/I/15/1/501826) to GS from the DBT - Wellcome Trust India Alliance. National Postdoctoral Fellowship (PDF/2016/002405) to DT from SERB, Department of Science and Technology, Govt. of India.

## Conflict of Interest

The authors declare that the research was conducted in the absence of any commercial or financial relationships that could be construed as a potential conflict of interest.

## Publisher’s Note

All claims expressed in this article are solely those of the authors and do not necessarily represent those of their affiliated organizations, or those of the publisher, the editors and the reviewers. Any product that may be evaluated in this article, or claim that may be made by its manufacturer, is not guaranteed or endorsed by the publisher.

## Supplementary Material

Supplementary Figure 1 Bar graph depicting the CHIKV genome copies **(A)** and DENV genome copies **(B)** in culture supernatants of CHIKV- or DENV-infected Huh7 cells at 24 and 36 h postinfection with 1 MOI. All the experiments were in done in three independent replicates, and the data shown are mean ± SEM. (*P < 0.05, ***P < 0.001)

Supplementary Figure 2 Bar graph depicting the CHIKV **(A)** and DENV **(B)** infectious virus titers estimated by foci-forming unit assay in the culture supernatants obtained from CHIKV- or DENV-infected cells, followed by UV treatment or no treatment. Bar graph depicting the CHIKV **(C)** and DENV **(D)** genome copies in cells infected for 24 h with the UV-treated or untreated culture supernatants described in A and B. All the experiments were in done in three independent replicates, and the data shown are mean ± SEM. (****P < 0.0001).

Supplementary Figure 3 Western blot analysis of FCγRII (CD32) in K562 cells infected with DENV preincubated with various dilutions of CHIKV patient sera S1 **(A)** and S4 **(B)**, CHIKV preincubated with various dilutions of DENV serotype 2 specific monoclonal antibody (mAb) **(C)** or DENV patient sera (DenS) **(D)**. Actin is used as internal protein loading control.

Supplementary Figure 4 Bar graphs depicting the densitometry analysis of the Western blots depicting the relative change in protein levels of p-TBK1 (S4A), TBK1 (S4B), p-IRF3 (S4C) and IRF3 (S45) with respect to mock-infected Huh7 cells. All experiments were done as three independent biological replicates and the data shown is +/- SEM. (ns, non-significant, *P < 0.05, **P < 0.01, ***P < 0.001).

Supplementary Table 1 Primers used for cloning about 500bp fragment from DENV or CHIKV genome spanning the primer binding sites used in qRT-PCR. The plasmid harboring the cloned fragment was used for the generation of standard curve for absolute quantification of viral genome copies.
