# Supplementary figures and images for: Comodulation of Dengue and Chikungunya Virus Infection During a Coinfection Scenario in Human Cell Lines
Source: Front Cell Infect Microbiol. 2022 Apr 28;12:821061. doi: 10.3389/fcimb.2022.821061 (PMC9097606; doi:10.3389/fcimb.2022.821061)

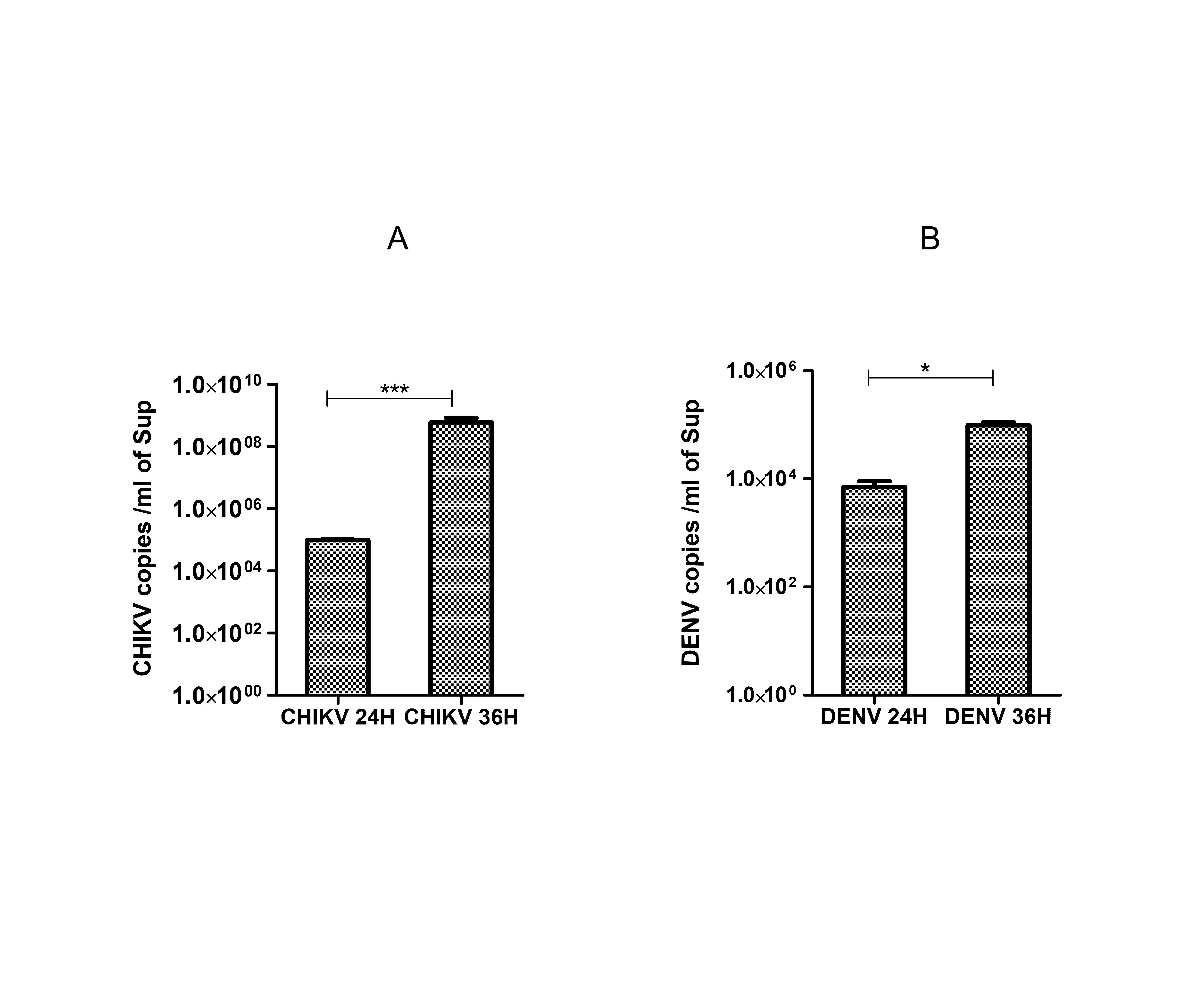

Supplement: Supplementary Figure 1 — Bar graph depicting the CHIKV genome copies (A) and DENV genome copies (B) in culture supernatants of CHIKV- or DENV-infected Huh7 cells at 24 and 36 h postinfection with 1 MOI. All the experiments were in done in three independent replicates, and the data shown are mean ± SEM. (*P < 0.05, ***P < 0.001) [file Image_1.jpeg]

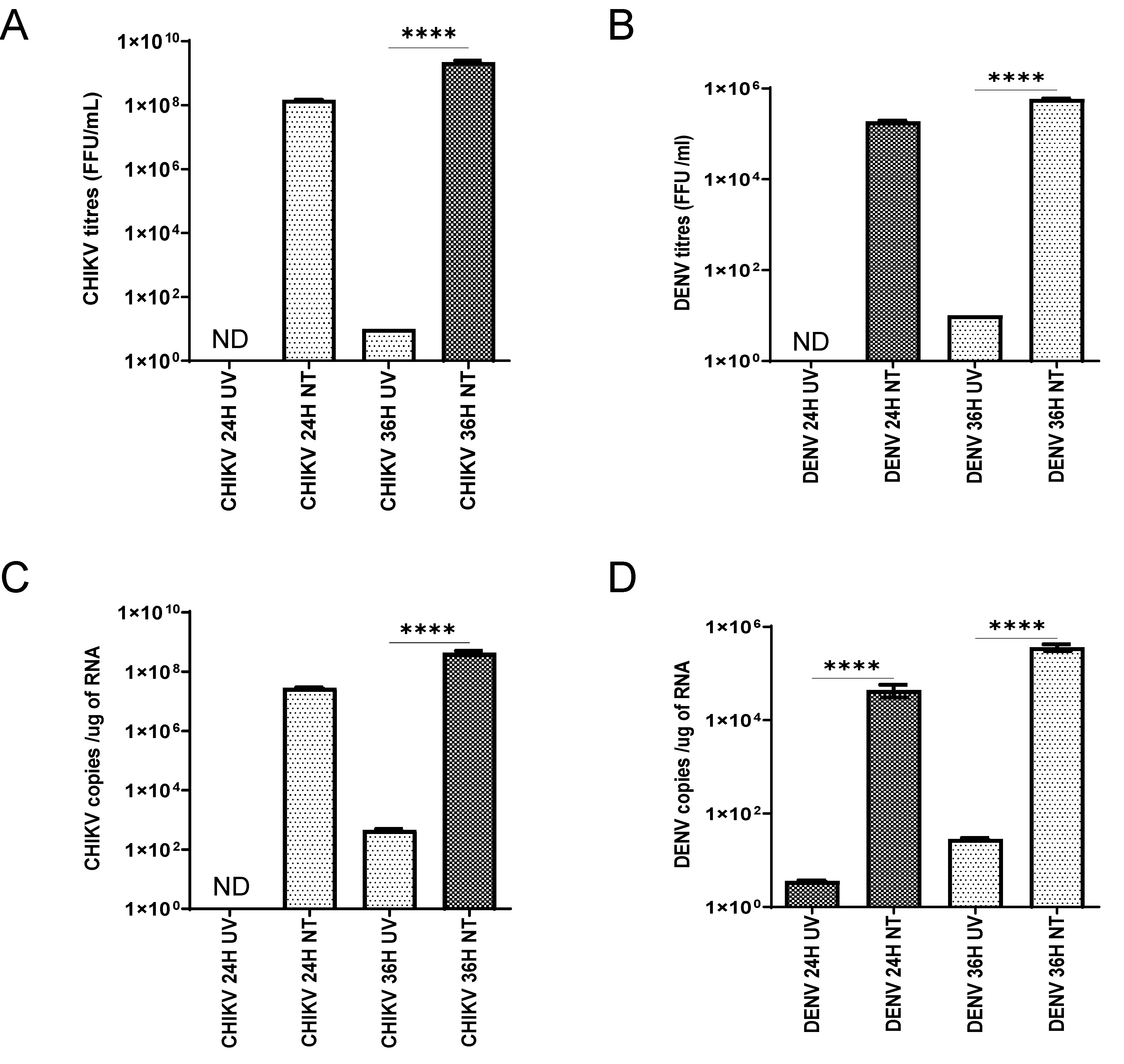

Supplement: Supplementary Figure 2 — Bar graph depicting the CHIKV (A) and DENV (B) infectious virus titers estimated by foci-forming unit assay in the culture supernatants obtained from CHIKV- or DENV-infected cells, followed by UV treatment or no treatment. Bar graph depicting the CHIKV (C) and DENV (D) genome copies in cells infected for 24 h with the UV-treated or untreated culture supernatants described in A and B. All the experiments were in done in three independent replicates, and the data shown are mean ± SEM. (****P < 0.0001). [file Image_2.tif]

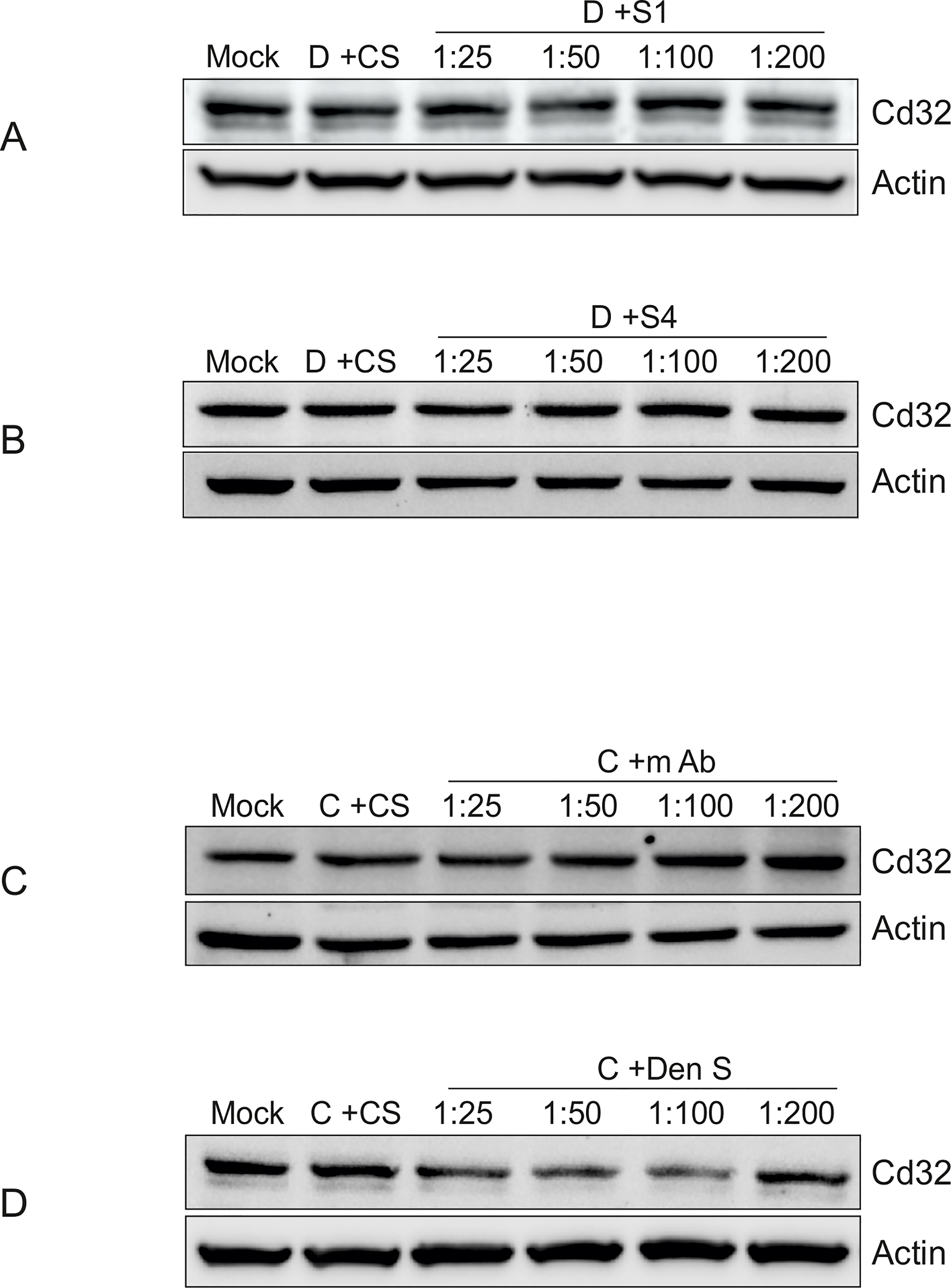

Supplement: Supplementary Figure 3 — Western blot analysis of FCγRII (CD32) in K562 cells infected with DENV preincubated with various dilutions of CHIKV patient sera S1 (A) and S4 (B), CHIKV preincubated with various dilutions of DENV serotype 2 specific monoclonal antibody (mAb) (C) or DENV patient sera (DenS) (D). Actin is used as internal protein loading control. [file Image_3.tif]

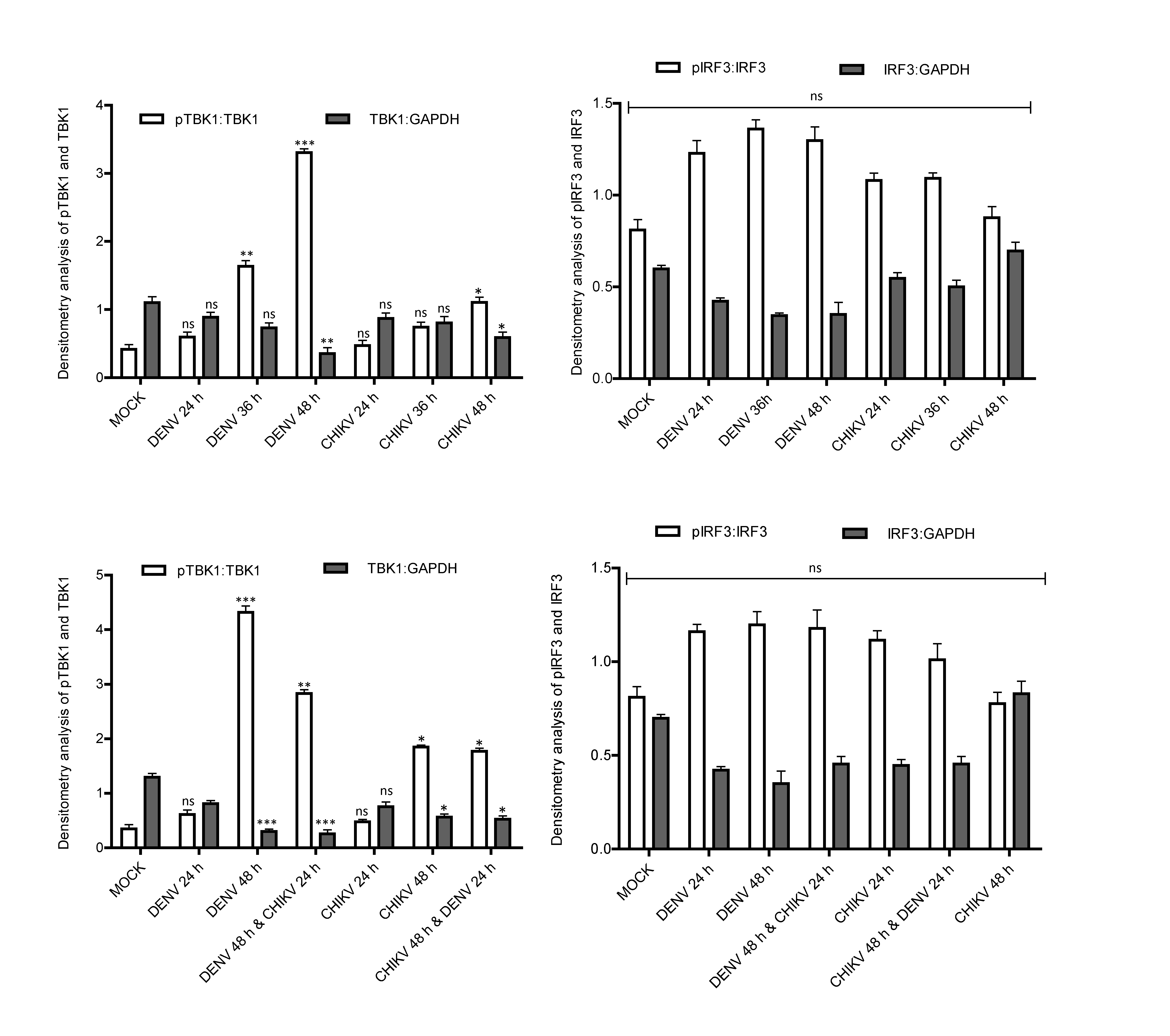

Supplement: Supplementary Figure 4 — Bar graphs depicting the densitometry analysis of the Western blots depicting the relative change in protein levels of p-TBK1 (S4A), TBK1 (S4B), p-IRF3 (S4C) and IRF3 (S45) with respect to mock-infected Huh7 cells. All experiments were done as three independent biological replicates and the data shown is +/- SEM. (ns, non-significant, *P < 0.05, **P < 0.01, ***P < 0.001). [file Image_4.tif]
