# Supplementary material for: Comodulation of Dengue and Chikungunya Virus Infection During a Coinfection Scenario in Human Cell Lines
Source: Front Cell Infect Microbiol. 2022 Apr 28;12:821061. doi: 10.3389/fcimb.2022.821061 (PMC9097606; doi:10.3389/fcimb.2022.821061)
Supplement: Supplementary Table 1 — Primers used for cloning about 500bp fragment from DENV or CHIKV genome spanning the primer binding sites used in qRT-PCR. The plasmid harboring the cloned fragment was used for the generation of standard curve for absolute quantification of viral genome copies. [file Table_1.docx]

| **Table S1: Primers used for cloning** | | | |
| --- | --- | --- | --- |
| **Primers** | **Primer sequence (5’-3’)** | **Position** | **Amplicon size** |
| **DENV2 FP** | GACGGCCAGTGAATTCAGTTGTTAGTCTACGTGGACCG | 1-22 | 500 bp |
| **DENV2 RP** | GCAGGTCGACTCTAGATTCCCTTTCTCTTGTCTGCTGACG | 500-477 |  |
| **CHIKV FP** | GACGGCCAGTGAATTCTGTAGTGGACACTACAGGCTCAACA | 2701-2725 | 500 bp |
| **CHIKV RP** | GCAGGTCGACTCTAGATGAAGGCTTGAATTATCTGGGACC | 3200-3177 |  |
